# Mass killing by female soldier larvae is adaptive for the killed male larvae in a polyembryonic wasp

**DOI:** 10.1038/s41598-019-43643-3

**Published:** 2019-05-14

**Authors:** Takahiro Otsuki, Daisuke Uka, Hiromu Ito, Genki Ichinose, Momoka Nii, Satoru Morita, Takuma Sakamoto, Maaya Nishiko, Hiroko Tabunoki, Kazuya Kobayashi, Kenji Matsuura, Kikuo Iwabuchi, Jin Yoshimura

**Affiliations:** 1 Department of Mathematical and Systems Engineering, Shizuoka University, Hamamatsu, 432-8561 Japan; 2 Faculty of Agriculture, Tokyo University of Agriculture and Technology, Fuchu, Tokyo 183-8509 Japan; 3 Department of International Health, Institute of Tropical Medicine, Nagasaki University, Nagasaki, 852-8523 Japan; 4 Department of Environmental Sciences, Zoology, University of Basel, 4051 Basel, Switzerland; 5 Hokkaido Forest Research Station, Field Science Education and Research Center, Kyoto University, Hokkaido, 088-2339 Japan; 6 Laboratory of Insect Ecology, Graduate School of Agriculture, Kyoto University, Kyoto, 606-8502 Japan; 7 Department of Environmental and Forest Biology, State University of New York College of Environmental Science and Forestry, Syracuse, NY 13210 USA; 8 Marine Biosystems Research Center, Chiba University, Kamogawa, Chiba 299-5502 Japan; 9 Present Address: Forestry promotion and Environment Department, Kochi Prefecture Office, Kochi, 780-0850 Japan

**Keywords:** Evolutionary theory, Behavioural ecology, Theoretical ecology, Entomology, Animal behaviour

## Abstract

Self-sacrifice is very rare among organisms. Here, we report a new and astonishing case of adaptive self-sacrifice in a polyembryonic parasitic wasp, *Copidosoma floridanum*. This wasp is unique in terms of its larval cloning and soldier larvae. Male clone larvae have been found to be killed by female soldier larvae, which suggests intersexual conflict between male and female larvae. However, we show here that mass killing is adaptive to all the killed males as well as the female soldiers that have conducted the killing because the killing increases their indirect fitness by promoting the reproduction of their clone sibs. We construct a simple model that shows that the optimal number of surviving males for both male and female larvae is very small but not zero. We then compare this prediction with the field data. These data agree quite well with the model predictions, showing an optimal killing rate of approximately 94–98% of the males in a mixed brood. The underlying mechanism of this mass kill is almost identical to the local competition for mates that occurs in other wasp species. The maternal control of the sex ratio during oviposition, which is well known in other hymenopterans, is impossible in this polyembryonic wasp. Thus, this mass kill is necessary to maximize the fitness of the female killers and male victims, which can be seen as an analogy of programmed cell death in multicellular organisms.

## Introduction

Self-sacrifice, or offering one’s own life, is a fairly rare and strange phenomenon among organisms, including humans. If natural selection favours individuals producing as many offspring as possible, sacrificing one’s own life seems impossible to evolve^1–4^. For example, an attempt to dive into a river to save a drowning child by an unrelated adult who cannot swim would lead to the loss of his/her direct fitness^1–4^. In some cases, however, self-sacrifice is truly adaptive^5^. Female cannibalism of a mating male partner is well known in mantises^6^, with male mantises often eaten by copulating females. This self-sacrifice is adaptive in terms of two aspects: (1) the male experiences successful copulation, and (2) he provides nutrition to his own offspring (eggs). Similar male sacrifice is also common in redback spider^7,8^, orb-weaving spider^9^ and many other spiders^10^.

Another prominent example of self-sacrifice is the defensive behaviours of honey bee workers^11–14^. The act of stinging in a honey bee results in death because the hooked stinger is connected to her own internal organs. Honey bees also form bee balls in defence against Asian giant hornets (*Vespa mandarinia*)^11,12^, and the bees forming the bee ball die because of the high temperature they create within the centre of the cluster of bees. These suicidal behaviours in honey bees represent colony defence, since a colony is considered to be a superorganism. Another yet surprising suicidal behavior is known in pea aphids that kill themselves when they are parasitized by a parasitic wasp^15,16^. The underlying mechanisms of such self-sacrifice in honey bee and pea aphids is explained by kin selection^1,5,11,15,17^.

**Figure 1:**
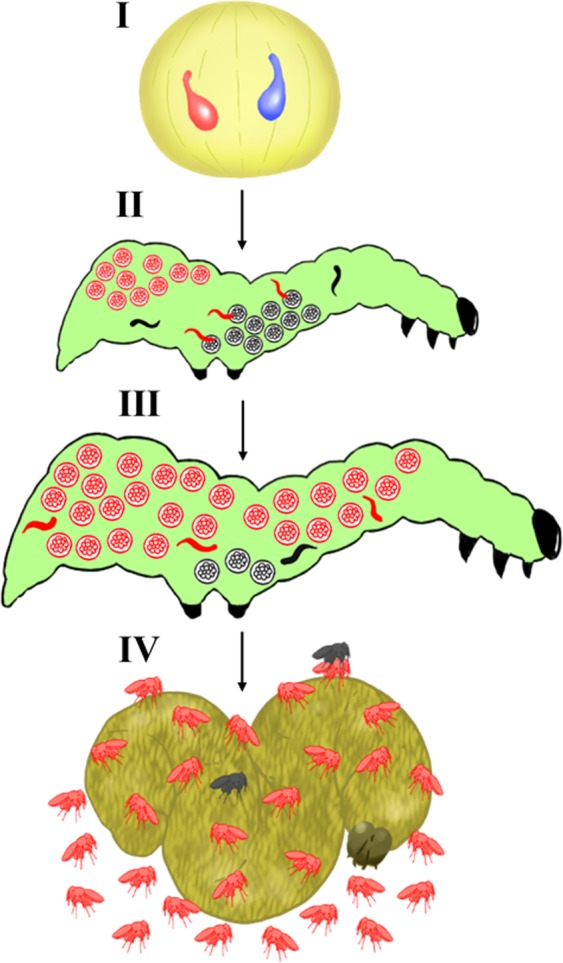
Schematic diagram of the process of development in a polyembryonic wasp. (**I**) A female wasp oviposits one fertilized egg (blue) and one unfertilized egg (red) in a host egg. (**II**) Male and female soldiers develop during embryo proliferation. Female soldiers attack male embryos. (**III**) The sex ratio of proliferating embryos is biased towards females. (**IV**) At emergence, a small number of male wasps mate with a large number of female wasps on or near the host mummy from which they have emerged.

Here, we report a unique case of adaptive self-sacrifice, the mass killing of male reproductive larvae by female soldier larvae in a polyembryonic parasitic wasp, *Copidosoma floridanum* (Hymenoptera, Encyrtidae) (Fig. 1). Mass kill is evidently adaptive for the female soldiers that kill the male larvae. Surprisingly, this mass kill is also adaptive for the killed males themselves. This wasp (*C. floridanum*) is unique in terms of its clonal reproduction (ca. 1,000–2,000 larvae from one egg) and the production of larval soldiers^18–20^. In *C. floridanum*, a female wasp will usually lay two eggs (one male and one female) on a host moth egg. Within a single host caterpillar, one wasp egg develops into 1,000–2,000 embryos (larvae), with most of them being reproductive and eventually developing into adult wasps that then emerge from the dead and desiccated caterpillar carcass. The rest become larval soldiers. These larval soldiers are known to attack competitive parasites that invade the host they inhabit. The killing of male reproductives by female soldiers is reported as sexual conflict between the males and females^18,21^. This killing is, however, suggested to be adaptive for the killed male reproductives because their clones can mate with the increased number of female wasps that can develop as a result of the space and nutrients made available in their host caterpillar as a result of their deaths^22^.

Grbić, Ode & Strand^22^ discovered the astonishing mass killing of male reproductives by female soldiers in a colony of *C. floridanum* in the USA. Because of the possibility of mating success after dispersal, they interpreted this phenomenon as sibling conflict (rivalry) between males and females. They also noted that the fitness of males is equivalent to that of females if there is no possibility of post-dispersal mating success in males^22^. We should note that, even if males can attain post-mating success, this success is extremely low compared with the success represented by mating with an additional female by his clone (see Supplementary Information for a numerical example). Because the sexual difference in body size in adult wasps is very small, we expect that one killed male can almost certainly be replaced by one additional female. In addition, because this female is usually a sister of the killed male, the killed male obtains higher inclusive fitness from the additional female than that from an unrelated female who is expected to mate with the considered male after dispersal. Therefore, a surviving male will have to locate multiple virgin females. However, the probability of finding a female-only colony is extremely low because most colonies (over 60%) are mixed colonies^22^. For example, if the post-dispersal mating success is 0.1%, then the successful male will have to mate with at least one thousand virgin females for his fitness to be barely equivalent to that of being killed. Thus, the additional fitness attained by post-dispersal mating is almost negligible, and the resulting fitness of males is almost equal to the number of females that emerge from the carcass. This means that the fitness of a male is equivalent to that of the female clones and their mother.

Here, we model the adaptive advantage of the killing of male reproductives. We then estimate the number of males killed in a single host in the field in Japan and evaluate the adaptive advantage of killed males in male mass kill by comparing the empirical data with the model predictions. We discuss this male mass kill in terms of male self-sacrifice, extraordinary sex ratios, programmed cell death, social evolution and related phenomena.

## Model

The model is as follows (Fig. 1). A female wasp lays two eggs, one female and one male, into a host egg. In the host egg, each polyembryonic wasp egg divides into 1,000–2,000 clone (genetically identical) embryos that develop into either reproductive larvae or soldier larvae. The reproductive larvae grow and eventually emerge from the host caterpillar carcass as adult wasps, while the soldier larvae do not emerge but are eventually digested by the host caterpillar before the emergence of the clone reproductives. We assume that the population of newly emerging adult wasps, *N* (from the larval clone reproductives), in a single host egg is constant, where *N*_F_ and *N*_M_ are the number of females and males, respectively: *N* = *N*_F_ + *N*_M_ = constant. We set *N* = 2,000. After adult emergence, each female instantly copulates with the surviving males on the host carcass. We do not consider the dispersion of females because the surviving males approach and copulate with the females immediately after their emergence^1,21,22^. Let *L* be the number of virgin females with which one male can copulate. When *L* = ∞ (infinity), one male can copulate with all the females emerging from the host carcass. Let *s*_M_ and *d*_M_ be the survival and mortality rates of the emerging males, respectively, such that *d*_M_ = 1 − *s*_M_, and *s*_F_ be the survival rate of females after dispersal until copulation.

Because of clonal reproduction in both the male and female offspring, the fitness of a mother wasp, all female clones and all male clones is the same and depends on the number of copulated females on the host carcass. We can ignore the survival rate *s*_F_ > 0 because it is independent of *N*_M_. The fitness of surviving larval males, *W*(*N*_M_, *d*_M_, *L*), is given by

1 $$W=W({N}_{{\rm{M}}},\,{d}_{{\rm{M}}},L)={\sum }_{k=1}^{{N}_{{\rm{M}}}}[{C}_{k}^{{N}_{{\rm{M}}}}{{s}_{{\rm{M}}}}^{k}{{d}_{{\rm{M}}}}^{{N}_{{\rm{M}}}-k}\,{\rm{\min }}\,\{kL,{N}_{{\rm{F}}}\}]\,$$

here $${C}_{k}^{{N}_{{\rm{M}}}}=C({N}_{{\rm{M}}},k)=(\begin{array}{c}{N}_{{\rm{M}}}\\ k\end{array})$$ means the number of *k*-combinations from *N*_M_ elements. The term $\sum _{k=1}^{{N}_{{\rm{M}}}}[{C}_{k}^{{N}_{{\rm{M}}}}{{s}_{{\rm{M}}}}^{k}{{d}_{{\rm{M}}}}^{{N}_{{\rm{M}}}-k}]$ is the expected numbers of surviving males to mate, and *kL* is the number of females that can be mated by *k* males. If the total number of females *N*_F_ is less than *kL*, then all *N*_F_ females are copulated by sib males; otherwise *kL* females becomes the number of females copulated by sib males (note that we assume that virgin females have no success). Thus *W* = *W*(*N*_M_, *d*_M_, *L*) is the total number of copulated females by sib males. Note that the overall fitness after one generation, *s*_F_*W*, can be measured in the field. When *L* = ∞, this equation reduces to

2 $$W=W({N}_{{\rm{M}}},\,{d}_{{\rm{M}}},\infty )=\,\{1-{{d}_{{\rm{M}}}}^{{N}_{{\rm{M}}}}\}{N}_{{\rm{F}}}$$

because one surviving male is sufficient to copulate with all emerging females. The purpose here is to find the optimal number of *N*_M_ when *W* is maximized, i.e., *W* → max.

## Result

**Figure 2:**
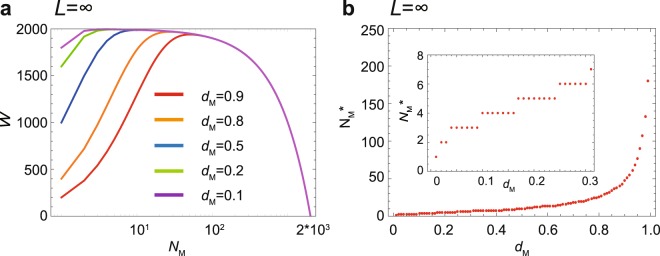
The optimal number of surviving males without male copulation limitation (*L* = ∞). (**a**) The fitness, *W*, as a function of *N*_M_ for a given mortality, *d*_M_. (**b**) The optimal number of surviving males, ${N}_{{\rm{M}}}^{\ast }$, against mortality, *d*_M_. Inset enlarges 0 ≤ *d*_M_ ≤ 0.3. *N* = 2000.

When a single male can copulate with all emerged females, i.e., *L* = ∞, the fitness, *W*, is a unimodal function of *d*_M_ (Fig. 2a). The optimal number of surviving males, ${N}_{{\rm{M}}}^{\ast }$, that maximizes *W* is an increasing function of *d*_M_ (Fig. 2b). Because the mortality of adult males, *d*_M_, is considered to be very small (*d*_M_ ≪ 0.3), ${N}_{{\rm{M}}}^{\ast } < 10$ (inset in Fig. 2b). The optimal number of surviving males, ${N}_{{\rm{M}}}^{\ast }$, is one at *d*_M_ = 0 and increases very slowly in a stepwise manner to 7 when *d*_M_ increases to 0.3. Thus, a small number of male adult wasps (${N}_{{\rm{M}}}^{\ast } < 10$) is sufficient for copulating with all emerging females in this wasp species. Note that the optimal number of surviving males at zero mortality, ${N}_{{\rm{M}}}^{\ast }$, is the round-up integer of [*N*/(*L* + 1)].

**Figure 3:**
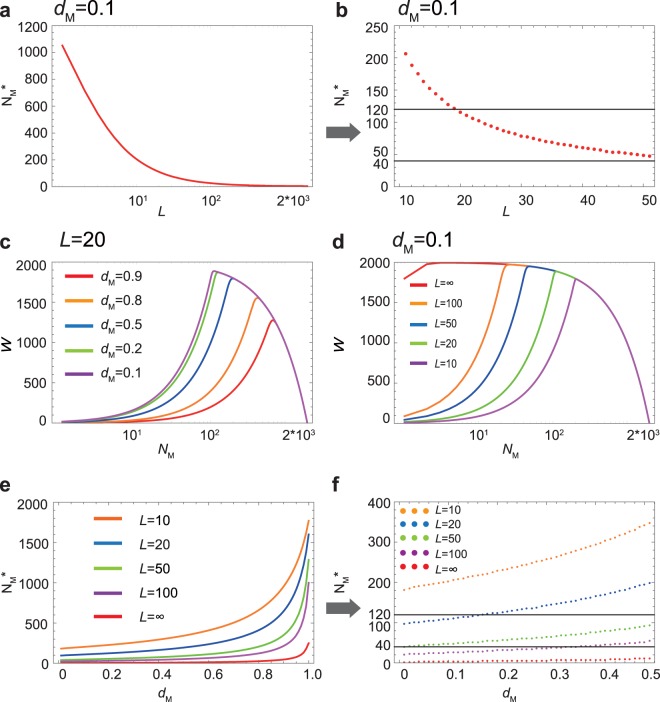
The optimal number of surviving males with varying levels of male copulation limitation. (**a**) The fitness, *W*, as a function of *L* for male mortality, *d*_M_ = 0.1. (**b**) An enlargement of (**a**) for *L* = 10~50. (**c**) The fitness, *W*, as a function of *N*_M_ for a given mortality, *d*_M_, when *L* = 20. (**d**) The fitness, *W*, as a function of *N*_M_ for a given copulation limitation, *L*, when *d*_M_ = 0.1. (**e**) The optimal number of surviving males, ${N}_{{\rm{M}}}^{\ast }$, against mortality *d*_M_ for various *L* = 10~∞. (**f**) An enlargement of (**e**) for 0 ≤ *d*_M_ ≤ 0.5. *N* = 2000. The observed ranges (*N*_M_ = 40~120) are indicated in (**b**) and (**f**).

A single male has a limited time available for copulation because many females emerge almost simultaneously. If there is a copulation limitation, *L*, for a male, then the optimal number of surviving males, ${N}_{{\rm{M}}}^{\ast }$, becomes greater than one (Fig. 3). When *L* decreases, the optimal male number, ${N}_{{\rm{M}}}^{\ast }$, increases (Fig. 3a,b), and when *L* = 10~50, ${N}_{{\rm{M}}}^{\ast }$ agrees well with the observed ranges (*N*_M_ = 40~120) at *d*_M_ = 0.1 (Fig. 3b). Note that the fitness function (Equation (1)) exhibits a more dramatic peak when *L* is introduced and decreases (Fig. 3c,d). The optimal number of surviving males, ${N}_{{\rm{M}}}^{\ast }$, also increases with *d*_M_ as in Fig. 2, but the initial value at *d*_M_ = 0.0 increases when *L* is decreased (Fig. 3e,f).

**Figure 4:**
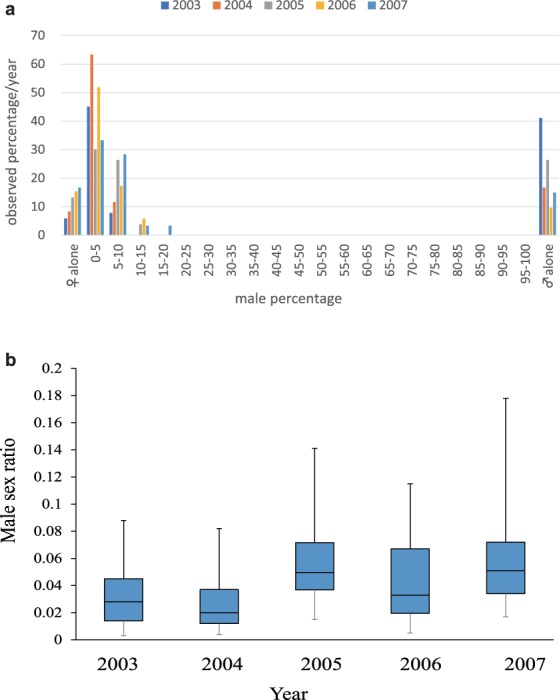
The annually observed male ratios in broods of *C. floridanum* collected in the field. (**a**) All data. No mixed broods (0%) had a male percentage of between 20 and 100%. (**b**) The proportions of emerging males in mixed broods only. Error bars indicate standard deviation. Data in 2003–2005 and 2007 were taken from Uka *et al*.^20^.

The data from the field and laboratory exhibit extraordinary sex ratios when the adults emerge from the host carcass (Fig. 4). The five-year experiment using laboratory-reared mixed broods shows that the adult sex ratios are approximately 0.02~0.06, indicating that there are 40~120 male wasps among the 2000 emerging wasps in total (Fig. 4a). The data from the field-collected carcasses also show that the percentage of males in most cases of mixed broods fell between 1 and 10%. Assuming the male mating ability *L* = 20~50 (Fig. 3b) and the male mortality range 0 ≤ *d*_M_ ≤ 0.3 (Fig. 3f), the expected optimal number of male wasps, ${N}_{{\rm{M}}}^{\ast }$, agrees quite well with the observed ranges (*N*_M_ = 40~120).

The current results show that being killed by a female soldier is adaptive for male reproductive clones (Figs 1–3). When a single male is assumed to be able to inseminate all the emerging females on a carcass, a very small number of males is sufficient to achieve the maximum fitness (Fig. 2). However, a single male is unlikely to be able to copulate with all the emerging females because of limitations in terms of time and sperm quantity. Therefore, we introduce a copulation limitation term, *L*, which represents the maximum number of females one male can inseminate (Fig. 3). When this limitation is incorporated into the model, the optimal number of males, ${N}_{{\rm{M}}}^{\ast }$, significantly increases. Assuming male mortality of *d*_M_ = 0.1 and a range of *L* of *L* = 10~50, the optimal number of surviving males falls between 40 and 120 individuals, which is well matched to the observed range of surviving males (Figs 3 and 4). We should note that our five years of field data from Japan not only confirmed the original discovery by Grbić *et al*.^22^ but also showed that the yearly male percentages were never greater than 20% in the mixed-sex broods (Fig. 4).

## Discussion

Our models are very simple and involve several important assumptions. We assume that a single mother wasp lays two eggs (one male and one female). However, even if two different mothers each lay one egg (of different sexes), the optimal number of surviving males is identical to that in the current results. We also ignore any successful mating after leaving the host carcass because such mating is very rare in this wasp compared with the mating that occurs on the carcass. In addition, we exclude mating competition with other males from different hosts because such opportunities should also be extremely rare. The observation of emerging wasps shows that mating is initiated by a male wasp immediately following his emergence from a carcass. We did not consider competition with other parasite species in the host, as such parasites will be killed by female larval soldiers. Because the killing of males is an additional function of female soldiers, a sufficient number of female soldiers is assumed to be present in a single carcass.

In this species, conflicts of interest concerning the sex ratio are not expected between the female killers and male victims^6^. If all male reproductives are killed, then the females must find a mate among the males emerging from other host carcass. The chance of finding such males is expected to be very low, resulting in the lack of mates for most emerging female wasps. Thus, from the perspective of both sexes, avoidance of the killing of all males should certainly be adaptive. There could be some protective mechanism in place to save a few male survivors; for example, soldier females may count or estimate the number of surviving males or at least save a few males from being killed. The male behaviour of escaping into fat bodies may be a part of such a protective mechanism.

Extremely female-biased sex ratios are often observed in fig wasps and parasitoid wasps in which mating take place among the offspring produced by one or a few mothers^22–25^. The model describing this situation, termed the local mate competition (LMC) theory, is functionally very similar to our model. In both models, the female-biased sex ratio reduces mating competition among sons while increasing the number of mating partners for the remaining sons. However, the sex ratio adjustment mechanisms are completely different in the two models. In the fig/parasitoid wasps, the adult sex ratio is generally adjusted by mothers at oviposition, while the sex-ratio adjustment is achieved by female soldiers (daughters) killing males within the host in the polyembryonic wasp^21^.

We should stress that the mothers of the polyembryonic wasp *C. floridanum* have no control over the sex ratio of the mixed brood. They lay only one or two eggs on a single host and avoid ovipositing in already parasitized hosts, while fig/parasitoid wasps lay multiple eggs at an optimal sex ratio according to the number of mothers on the fig/host. The mothers have no information about the optimal number of reproductive males in a mixed brood because it is determined during the development in the host larvae^22^. The optimal number of reproductive males depend the total capacity (size) of a host larvae (carcass), varying more than two-folds. Futhermore, if the female egg is removed or ceased to develop, then the male egg should have no need for reducing the number of reproductive males. Thus, even if the mothers have an ability to control the number of male offspring, they cannot know the optimal number of males that will be determined at a much later stage. This is the difference between the current polyembryonic wasp and the fig wasps.

This difference might have arisen from the primary function of female soldiers of killing all potential competitors’ eggs that are laid later will be killed by the soldiers in the host. A similar situation occurs in other parasitoid wasps (*Melittobia* spp.), in which lethal male-male combat results in an extreme female-biased sex ratio regardless of the number of mothers^26,27^. The females of *Melittobia* spp. lay female eggs in already infested hosts because lethal combat occurs only between males^26^, which indicates that lethal combat is avoided through the sex ratio adjustment caused by the mothers. In the polyembryonic wasp *C. floridanum*, the mass killing is unavoidable to achieve the optimal sex ratio for both males and females due to their particular development, polyembryony.

Kin selection (inclusive fitness) and polyembryonic cloning are the necessary mechanisms leading to the adaptive mass killing of male clone reproductives in this species. Kin selection can explain the self-sacrifice of reproduction/life to facilitate the survival of close kin^17^. The mass killing of males addressed in this paper can be viewed as an extreme case of kin selection through the act of being killed, as the males sacrifice their lives, preventing them from developing into the adult stage. Even though most of the males are killed eventually, they often exhibit escape behaviour by hiding in a fat body, likely because some of them need to survive. Thus, the maximization of fitness is assured by being killed, and this condition is only possible in polyembryonic wasps. Here, the colony of all wasp larvae in a single host caterpillar is considered to be a single superorganism, similar to the colonies of various bees, wasps and ants. In this sense, the phenomenon of self-sacrifice is analogous to programmed cell death (PCD), where PCD is defined as “adaptation for death triggered by abiotic or biotic environmental stresses” (ref.^28^)^5^.

Here, we report another unique case of adaptive self-sacrifice based on kin selection in *C. floridanum*, in which being killed by female soldiers (not committing suicide) is adaptive for the reproductive males themselves. This phenomenon is related to kin selection, polyembryony, cloning, eusociality, after-birth sex ratio control, extraordinary sex ratios, adaptive suicidal behaviour and self-sacrifice. Initially, the male reproductive larvae killed via mass kill appear to be forced to die without any benefit to them. However, this kill is adaptive for the killed males because of the increased success of their male clones. Thus, the existence of polyembryonic cloning and larval soldiers in this species has enabled the development of this exceedingly unique phenomenon: being killed is adaptive to the killed individuals, which is a life history strategy that has never before been seen in the organismal world.

## Supplementary information

Supplementary Information

## Data Availability

The authors declare that all data supporting the findings of this study are available within the article and its Supplementary Information files or from the corresponding author upon reasonable request.
