# Supplementary material for: Mass killing by female soldier larvae is adaptive for the killed male larvae in a polyembryonic wasp
Source: Sci Rep. 2019 May 14;9:7357. doi: 10.1038/s41598-019-43643-3 (PMC6517382; doi:10.1038/s41598-019-43643-3)
Supplement: Supplementary file 1 — Supplementary Information [file 41598_2019_43643_MOESM1_ESM.pdf]

Supplementary Information

Mass killing by female soldier larvae is adaptive for the killed male larvae in a polyembryonic wasp

Takahiro Otsuki[1,#] Daisuke Uka[2,#,$], Hiromu Ito[3,4], Genki Ichinose[1], Momoka Nii[1], Satoru Morita[1], Takuma Sakamoto[2], Maaya Nishiko[2], Hiroko Tabunoki[2], Kazuya Kobayashi[5], Kenji Matsuura[6], Kikuo Iwabuchi[2, *] and Jin Yoshimura[1,7,8, *]

[1] Department of Mathematical and Systems Engineering, Shizuoka University, Hamamatsu, 432-8561, Japan

[2] Faculty of Agriculture, Tokyo University of Agriculture and Technology, Fuchu, Tokyo 183-8509, Japan

[3] Department of International Health, Institute of Tropical Medicine, Nagasaki University, Nagasaki 852-8523, Japan

[4] Department of Environmental Sciences, Zoology, University of Basel, 4051 Basel, Switzerland

[5] Hokkaido Forest Research Station, Field Science Education and Research Center, Kyoto University, Hokkaido 088-2339, Japan

[6] Laboratory of Insect Ecology, Graduate School of Agriculture, Kyoto University, Kyoto 606-8502, Japan

[7] Department of Environmental and Forest Biology, State University of New York College of Environmental Science and Forestry, Syracuse, NY 13210 USA

[8] Marine Biosystems Research Center, Chiba University, Kamogawa, Chiba 299-5502, Japan

[$]Present address: Forestry promotion and Environment Department, Kochi Prefecture Office, Kochi 780-0850, Japan

[#]these authors contributed equally.

*****Correspondence and requests for materials should be addressed to J.Y. (yoshimura.jin@shizuoka.ac.jp) or to K.I. (kikkuo@cc.tuat.ac.jp).

1. A numerical example of the inferiority of post-dispersal mating in males

We consider that a male has a two choices: (1) acceptance of his own death due to killing by a female soldier larva or (2) escape from being killed to become an adult, with subsequent dispersal to a new, female-only colony to mate with unrelated females. Here, all females from his own mixed colony (including one female replacement as a result of his death) have been mated by his male clones. If he escapes killing, he will prevent his replacement with a female clone that could have been mated by one of his male clones. Thus, he can increase his probability of mating with unrelated females emerging from a female-only colony but will sacrifice the survival of one female sibling that could be mated by his clone. Note that mating by emerging males occurs immediately after females emerge from the carcass in the mixed colonies. The unmated males have to seek out unrelated females emerging from female-only colonies (approximately 10% of all colonies; see Fig. 4)[5]. Approximately 20% of all colonies contain only males (see Fig. 4)[5], and all the males from these male-only colonies disperse to search for females emerging from a female-only colony immediately after emergence. This means that an unmated male from a mixed-brood colony must compete for females with a vast number of male wasps from male-only colonies. Note that each male-only colony produces approximately 2000 adult males.

We thus consider which choice is advantageous: (1) accept death and be substituted with a mated female sibling or (2) avoid death and attempt post-dispersal mating with unrelated females. If the latter is better, sibling rivalry or conflict may have occurred, as suggested by Grbić et al.[5]. Here, we show that the occurrence of the latter is negligible compared to that of the former.

The comparison is as follows. Let N_{M}, N_{F} and N be the number of males, females and all individuals, respectively, such that $N_{\mathrm{M}} + N_{\mathrm{F}} = N$. Suppose that L is the number of females on the host carcass with which a single male can copulate. We now consider the choice of a single male in N_{M}, where if he stays to be killed, he is replaced by a female sibling. Here, all females are copulated by the remaining clone males, such that $(N_M\text{-}1)L >= N_{\mathrm{F}}+1$. Therefore, if he stays, the total number of the copulated siblings increases by one. However, if he disperses, the total number of copulated siblings stays the same, i.e., N_F. The possible benefit is only mating with unrelated females from a female-only colony. We can summarize the comparison of the two choices as follows:

Choice 1 (Stay): 1 copulated sibling female

Choice 2 (Leave): the expected mating with unrelated females

Let P_S be the probability of success in finding and mating with females on a female-only carcass. Then, Choice 2 becomes $P_S *L$. If this choice is better than Choice 1, we get

$$P_S *L > 1.$$

Simply assuming $L = 100$, we get $P_S > 0.01$. However, the probability of mating success is expected to be extremely low because the number of female-only colonies is only approximately 10% of the total number of colonies (see Fig. 4)[5]. In addition, the number of male-only colonies is approximately 20% of the total, and all males from those colonies are searching for female-only colonies (see Fig. 4)[5]. The maximum number of females that can be copulated by a single male is expected to be much lower, since each copulation takes time. Furthermore, the relatedness of the offspring produced by this mating is lower, roughly $(1/2) P_S *L > (5/8)*1$, because for a clone male the relatedness of his sibling female is 0.5 under haplodiploidy. Note that for a female clone the relatedness of her sibling male is 0.25 in these hymenopterans.

Thus, because the probability of post-dispersal success is less than 1%, the attempt to survive and disperse is never a better choice than staying to be killed.

2. Methods

The polyembryonic parasitoid *C. floridanum* and its larval host *Ctenoplusia* (=*Acanthoplusia*) *agnata* (Lepidoptera, Noctuidae) were collected from burdock fields in Fuchu and Koganei (ca. 3 km apart), Tokyo, Japan, and the hosts were reared in the laboratory. We observed whether copulation was attempted when the wasps were emerging from a carcass. If no copulation attempts were identified, we examined one hundred individuals to sex the broods. Copulation attempts were always observed in mixed broods. When attempted copulation was observed, we sexed one thousand individuals to estimate the emerging sex ratios of those mixed broods.

Supplementary Table S1. List of parameters.

Term	Range	Description
$\boldsymbol{L}$	$1 \le L \le \infty$	**Number of virgin females that one male can copulate with**
$\boldsymbol{N}$	$N = N_M + N_F$	**Population of emerging adult wasp** $(N = \text{Const.} = 2000)$
$\boldsymbol{N_M}$		**Number of males**
$\boldsymbol{N_M^*}$		**The optimal number of survived males**
$\boldsymbol{N_F}$		**Number of females**
$\boldsymbol{d_M}$	$0 \le d_M < 1$	**Mortality rate of emerging males**
$\boldsymbol{s_M}$	$0 < s_M \le 1$	**Survival rate of emerging males**
$\boldsymbol{s_F}$	$s_F > 0$	**Survival rate of emerging females (independent of** N_M**)**
$\boldsymbol{W}$		**The fitness of surviving larval males**
$\boldsymbol{P_S}$	$0 \le P_S \le 1$	**Probability of success in finding and mating with females from a female-only colony**

3. Field Data

Supplementary Table S2. The proportion of mix/pure broods in the examined field samples.

	♂ brood	♀ brood	Mix brood	Total Number
2003	21	3	27	51
2004	10	5	45	60
2005	14	7	32	53
2006	5	8	39	52
2007	9	10	41	60

Supplementary Table S3. The male ratios of mix pure broods in the examined field samples.

2003			2004			2005			2006			2007		
♂	♀	♂ratio	♂	♀	♂ratio	♂	♀	♂ratio	♂	♀	♂ratio	♂	♀	♂ratio
3	992	0.003	4	996	0.004	15	985	0.015	5	995	0.005	17	983	0.017
6	994	0.006	5	995	0.005	19	981	0.019	7	993	0.007	19	981	0.019
6	994	0.006	6	994	0.006	20	980	0.020	8	992	0.008	21	979	0.021
7	993	0.007	6	994	0.006	22	978	0.022	8	992	0.008	25	975	0.025
11	989	0.011	7	993	0.007	25	975	0.025	10	990	0.010	25	975	0.025
12	988	0.012	9	991	0.009	30	970	0.030	11	989	0.011	28	972	0.028
13	987	0.013	9	991	0.009	36	964	0.036	15	985	0.015	29	971	0.029
15	985	0.015	10	990	0.010	36	964	0.036	16	984	0.016	29	971	0.029
16	984	0.016	10	990	0.010	37	963	0.037	18	982	0.018	31	969	0.031
17	983	0.017	11	989	0.011	38	962	0.038	19	981	0.019	33	967	0.033
19	971	0.019	11	989	0.011	40	960	0.040	20	980	0.020	34	966	0.034
21	979	0.021	12	988	0.012	42	958	0.042	21	979	0.021	35	965	0.035
21	951	0.022	12	988	0.012	43	957	0.043	23	977	0.023	36	964	0.036
28	972	0.028	14	986	0.014	48	952	0.048	23	977	0.023	38	962	0.038
31	969	0.031	15	985	0.015	48	952	0.048	26	974	0.026	39	961	0.039
32	968	0.032	17	983	0.017	49	951	0.049	28	972	0.028	39	961	0.039
34	968	0.034	17	983	0.017	50	950	0.050	31	969	0.031	40	960	0.040
38	962	0.038	17	983	0.017	54	946	0.054	31	969	0.031	47	953	0.047
41	959	0.041	18	982	0.018	59	941	0.059	32	968	0.032	48	952	0.048
43	957	0.043	19	981	0.019	62	938	0.062	33	967	0.033	49	951	0.049
47	953	0.047	19	981	0.019	63	937	0.063	35	965	0.035	51	949	0.051
48	952	0.048	20	980	0.020	65	935	0.065	38	962	0.038	53	947	0.053
49	951	0.049	20	980	0.020	68	932	0.068	40	960	0.040	54	946	0.054
50	950	0.050	21	979	0.021	71	929	0.071	41	959	0.041	59	941	0.059
52	948	0.052	22	978	0.022	73	927	0.073	44	956	0.044	59	941	0.059
68	932	0.068	22	978	0.022	79	921	0.079	44	956	0.044	61	939	0.061
88	912	0.088	26	974	0.026	85	915	0.085	47	953	0.047	65	935	0.065
			28	972	0.028	88	912	0.088	63	937	0.063	68	932	0.068
			29	971	0.029	89	911	0.089	64	936	0.064	68	932	0.068
			30	970	0.030	97	903	0.097	70	930	0.070	69	931	0.069
			31	969	0.031	115	885	0.115	71	929	0.071	72	928	0.072
			32	968	0.032	141	859	0.141	72	928	0.072	75	925	0.075
			33	967	0.033				74	926	0.074	78	922	0.078
			37	963	0.037				84	916	0.084	80	920	0.080
			38	962	0.038				88	912	0.088	80	920	0.080
			40	960	0.040				98	902	0.098	88	912	0.088
			41	959	0.041				100	900	0.100	89	911	0.089
			43	957	0.043				112	888	0.112	101	899	0.101
			50	950	0.050				115	885	0.115	133	867	0.133
			59	941	0.059							154	846	0.154
			61	939	0.061							178	822	0.178
			62	938	0.062									
			70	930	0.070									
			81	919	0.081									
			82	918	0.082									

Supplementary Table S4. The count data for each male-percentage class of broods in the examined field samples (data for Fig. 4a).

count																						
	(♀alone)0	0-1	1-5	5-10	10-15	15-20	20-25	25-30	30-35	35-40	40-45	45-50	50-55	55-60	60-65	65-70	70-75	75-80	80-85	85-90	90-95	95-100	(♂alone)100	Total number
2003	3	4	19	4	0	0	0	0	0	0	0	0	0	0	0	0	0	0	0	0	0	0	21	51
2004	5	7	31	7	0	0	0	0	0	0	0	0	0	0	0	0	0	0	0	0	0	0	10	60
2005	7	0	16	14	2	0	0	0	0	0	0	0	0	0	0	0	0	0	0	0	0	0	14	53
2006	8	4	23	9	3	0	0	0	0	0	0	0	0	0	0	0	0	0	0	0	0	0	5	52
2007	10	0	20	17	2	2	0	0	0	0	0	0	0	0	0	0	0	0	0	0	0	0	9	60

ratio																						
	(♀alone)0	0-1	1-5	5-10	10-15	15-20	20-25	25-30	30-35	35-40	40-45	45-50	50-55	55-60	60-65	65-70	70-75	75-80	80-85	85-90	90-95	95-100	(♂alone)100
2003	5.9	7.8	37.3	7.8	0.0	0.0	0.0	0.0	0.0	0.0	0.0	0.0	0.0	0.0	0.0	0.0	0.0	0.0	0.0	0.0	0.0	0.0	41.2
2004	8.3	11.7	51.7	11.7	0.0	0.0	0.0	0.0	0.0	0.0	0.0	0.0	0.0	0.0	0.0	0.0	0.0	0.0	0.0	0.0	0.0	0.0	16.7
2005	13.2	0.0	30.2	26.4	3.8	0.0	0.0	0.0	0.0	0.0	0.0	0.0	0.0	0.0	0.0	0.0	0.0	0.0	0.0	0.0	0.0	0.0	26.4
2006	15.4	7.7	44.2	17.3	5.8	0.0	0.0	0.0	0.0	0.0	0.0	0.0	0.0	0.0	0.0	0.0	0.0	0.0	0.0	0.0	0.0	0.0	9.6
2007	16.7	0.0	33.3	28.3	3.3	3.3	0.0	0.0	0.0	0.0	0.0	0.0	0.0	0.0	0.0	0.0	0.0	0.0	0.0	0.0	0.0	0.0	15.0
